# Production of sealed rod sources made from epoxy resin for the Saint-Petersburg brick phantom for the calibration of whole-body counters

**DOI:** 10.1007/s00411-022-00987-1

**Published:** 2022-07-14

**Authors:** Patrick Woidy, Oliver Meisenberg

**Affiliations:** Federal Office for Radiation Protection, Medical and Occupational Radiation Protection, Ingolstädter Landstraße 1, 85764 Oberschleißheim, Germany

**Keywords:** Internal monitoring, Whole-body counting, Anthropomorphic phantom, Sealed radioactive source, Epoxy resin

## Abstract

Rod sources are a common tool for the calibration of whole-body counters in combination with the Saint-Petersburg brick phantom. Here, a method for the production of such sources in ordinary radiochemical laboratories is presented. The rod sources consist of a tubular capsule of rigid polyvinyl chloride with a radioactive filling of epoxy resin. The method allows the production of rod sources at material costs of about 1 € per rod source and of ten rod sources by one person per day. Quality-assurance measurements were performed regarding the spatial distribution of the activity within the rod sources and the distribution of the activity throughout a set of sources. The relative double standard deviation of the activities of five different segments of single rod sources was 7.1%. The relative double standard deviation within a set of 90 rod sources was 2.8% after those 11% of sources with the greatest deviation from the arithmetic mean were discarded. Tests according to ISO 2919 to certify the rod sources as sealed sources of Class 2 of this standard were successfully conducted. The bending test proved to be the most critical test for the rod sources; the sources were broken by a mass of 12–14 kg, which is only slightly more than the stipulated mass of 10.2 kg. The presented method allows for a cost- and labour-effective production of sealed radioactive rod sources and thus facilitates the application of the Saint-Petersburg brick phantom for calibrations and interlaboratory comparisons of whole-body counters.

## Introduction

Whole-body counters are measurement devices used to determine the activity of photon-emitting radionuclides with homogeneous spatial distribution in the human body. They are usually calibrated with anthropomorphic phantoms that simulate the shape of the human body. Such phantoms are made from material with sufficiently similar properties to human tissues regarding the attenuation of photon radiation, and can be equipped with suitable radionuclides of known activity. A common type of such a phantom is the Saint-Petersburg brick phantom or universal phantom UPh (also called Igor, Olga or Irina in various countries; ICRU 1992; Kovtun et al. 2000). This phantom consists of blocks made of high-density polyethylene, which can be assembled in different layouts to simulate different physiques and postures of the human body. Each brick includes several drill holes (typically two or four), which can be equipped with solid rod-shaped radionuclide sources. The phantom features tissue-equivalent properties regarding the attenuation of photon radiation at least in an energy range between 150 and 3000 keV (Kovtun et al. 2000).

The rod sources for the Saint-Petersburg brick phantom have a length of approximately 16.5 cm and a diameter of 6 mm. The rod sources provided by several producers have in common that they consist of a tubular capsule of inactive material, the radioactive solid filling, and inactive plugs on both ends of the capsule. Different makeups comprise:

- Capsules of polypropylene and radioactive fillings of powdery ion-exchange resin (Scientific Research Institute for Industrial and Sea Medicine, Russia; Kovtun et al. 2000);
- Capsules of acrylic glass and radioactive fillings of epoxy resin (Eckert & Ziegler Nuclitec, Germany);
- Outer capsules of polyvinyl chloride, inner capsules of polyethylene and radioactive fillings of gypsum (for long-lived radionuclides) and capsules of polyvinyl chloride and radioactive fillings of agar–agar (for short-lived radionuclides; Radiochemistry Munich, Germany; Anonymous 2014);
- Capsules of flexible, rubber-like synthetic material and radioactive fillings of epoxy resin (IRSN, France; Benyakoub 2017);
- Capsules of polyethylene and radioactive fillings of epoxy resin (Institute of Radiation Physics, Lausanne, Switzerland; Bailat et al. 2014).

In the following, a novel method for the production of such rod sources with various radionuclides is presented in detail and the metrological quality of the rod sources is discussed. This method is similar to that developed by the Institute of Radiation Physics, Lausanne, and is feasible for producing such sources in general radiochemical laboratories. The rod sources comply with the requirements for sealed radioactive sources according to ISO 2919 (ISO 2012), which is a useful feature regarding their applicability.

## Materials and methods

### Production of the rod sources

The produced rod sources consist of tubes of rigid polyvinyl chloride as the inactive capsule, a filling of epoxy resin that is blended with a radionuclide solution, and one plug of inactive epoxy resin at each end of the tube. Consumables used for the production of the rod sources are presented in Table 1.

**Table 1:** Consumables used for the production of the rod sources

No	Consumable	Details	Manufacturer	Required quantity for 100 rod sources (including discard)
1	Tubes	Rigid PVC, transparent colourless, impact-resistant, length 162.0 mm (tolerance ± 0.3 mm), inner diameter 5.4 mm (tolerance ± 0.3 mm), outer diameter 6.0 mm (tolerance − 0.2 mm, + 0.0 mm)	K. + C. Weiss, Germany	100
2	Epoxy resin	“Ep-Gießharz wasserklar und Härter” (epoxy pouring resin, limpid, and hardener)	R&G Faserverbundwerkstoffe, Germany	350 g
3	Paint for identification of the sources	Enamel model-making paint	Revell, Germany	20 ml
4	Mixing vessels	Transport tubes, self-standing, with screw cap and conical bottom, 5 ml	Axygen Scientific, USA	100 for the filling, ≈ 10 for the plugs
5	Pipette tips for	Filter tips, of plastic, with aerosol barrier; volumes:	VWR International, USA	
	Hardener	1000 µl		≈ 20
	Radionuclide solution	100 µl		100
	Radioactive filling	1000 µl		100
	Second plug in combination with an air displacement pipette	1000 µl		≈ 20
6	Pipette tips for viscous media (for the transfer of inactive epoxy resin into the mixing vessel) in combination with a repetitive pipette^a^	ViscoTips 10 ml	Eppendorf, Germany	≈ 20
7	Pipette tips for viscous media (for the production of the first plug) in combination with a repetitive pipette^a^	CombiTips advanced 5 ml	Eppendorf, Germany	≈ 20

^a^A repetitive pipette was used so that material could be dispensed into several mixing vessels or for several plugs one after the other from one filling of the pipette with a larger amount of epoxy resin. As compared to an air-displacement pipette, this facilitates the work significantly at the expense of precision of dispensed material, which is not required during these two steps though

The production comprised the following steps:

1. Production of the first plug: an adhesive plastic label with the identification of the rod source was inserted into the tube and stuck to its inner surface. The end of the tube was tightly sealed with Parafilm and the tube was positioned in vertical orientation with the open side up in a mount. 1.0 ml of hardener and 3.0 ml of epoxy resin were pipetted into a mixing vessel. The aspiration into the pipette must be conducted slowly to avoid the emergence of air bubbles. A maximum of 3 drops of model-making paint were added to the hardener to identify the type of rod source by the colour of its plugs. The mixture was blended with a vortex mixer. The blend was left in the mixing vessel for at least 5 min to reduce the number of air bubbles that emerged during blending. After having pipetted 300 µl of the blend into the tube from the open side, the blend ran down the tube onto the Parafilm. The plug produced in that way had a length of about 13 mm. The blend was left for hardening for at least 2 days and the Parafilm was subsequently removed. Several plugs were produced from the same blend one after the other.

2. Preparation of the filling: 0.8 ml of hardener, the required amount of radionuclide solution (in the order of magnitude of 10–100 µl) and 2.4 ml of epoxy resin were pipetted into a mixing vessel and blended for 2 min using a vortex mixer. This must be done carefully so that no material hits the cap of the mixing vessel. After waiting for 5 min to reduce the number of air bubbles, the blend was pipetted into the tube in several portions. This was done slowly so that the blend ran down the inner side of the tube without plugging it. The hardening took 2 days.

The mixing vessel was weighed with a laboratory balance (CP124S with draught shield, resolution 0.1 mg, Sartorius, Germany) before adding the components (mass *m*_1_), after adding the hardener (mass *m*_2_), after adding the radionuclide solution (*m*_3_), after adding the epoxy resin (*m*_4_), and at the end of the process to determine the amount of blend that remained in the mixing vessel (*m*_5_). The mass *m*_r_ of the radionuclide solution in the tube, which can be used for the calculation of the activity of the rod source, can be calculated according to Eq. 1.

1 $${m}_{\text{r}}= \left({m}_{3}-{m}_{2}\right)\cdot \frac{{m}_{4}-{m}_{5}}{{m}_{4}-{m}_{1}}$$

Where the first factor is the mass of radionuclide solution in the blend, and the second factor is the fraction of the blend that is pipetted into the tube. The amount of radionuclide solution that remained in the pipette tip was negligible. It is recommended to certify the activity of the rod sources not only from the mass of radionuclide solution but also based on measurements of the rod sources on a gamma-spectroscopy detector.

3. Production of the second plug and enhancement of the first plug: the second plug was pipetted from an inactive blend of epoxy resin and hardener with the blend filled exactly up to the rim of the tube. After hardening of this plug, the rod source was taken from its mount. An additional drop of inactive blend was pipetted on the first plug (whose outer surface featured a concave, inwardly curved shape) so that a convex, outwardly curved surface originated; this enhanced the robustness of the rod source against pressure on the front surface. Care was taken that no blend flowed down the outer side of the tube during this step.

### Quality-assurance measurements

For a proof of concept, measurements were conducted with a set of 90 rod sources of ^137^Cs with an activity of about 80 Bq each. It is noted that similar measurements were conducted with every set of rod sources that was produced so far.

The activity of single rod sources was measured with a high-purity germanium (HPGe) gamma-spectroscopy detector (GMX series, n-type, Ortec, USA) with a crystal diameter of 74 mm and relative efficiency of 76%. The detector was calibrated with rod sources of ^60^Co, ^133^Ba and ^137^Cs (Eckert & Ziegler Nuclitec GmbH, Germany). The activity of these sources is traceable to a primary standard and the manufacturer is accredited as a calibration laboratory. The produced ^137^Cs rod sources were centred one after the other directly on the plastic cover of the end cap of the detector. Their activities were calculated using the detector efficiency at an energy of 662 keV obtained from the ^137^Cs calibration measurement. The projection of the rod sources over the edge of the detector did not matter because of the homogeneous spatial distribution of the activity within the single rod sources, which is explained in the following. Measurements of nuclides that were not present in the calibration standard were performed with a geometry with smaller counting efficiency, to reduce the effect of cascade summing.

Additionally, comparative measurements were conducted to check the spatial homogeneity of the activity along the rod sources. For this purpose, a rectangular collimator was mounted above the detector. The collimator was made of lead with a thickness of 4 cm and had an opening with a width of 3 cm, through which five slightly overlapping 3 cm segments of each rod source (with approximately 14 cm of radioactive filling between the plugs) were separately measured. Results were compared with each other by their count rates. Although possible spatial inhomogeneities within the rod sources would hardly matter in the measurement of entire phantoms, homogeneous activity distributions are crucial for the correct evaluation of the quality-assurance measurements, in particular of those with close contact between rod source and detector surface.

Uncertainties are presented with a coverage factor of *k* = 2.

### Tests for the classification as sealed radioactive sources

Tests according to ISO 2919 were conducted to qualify the rod sources as sealed sources. ISO 2919 stipulates a variety of tests, each with different levels of severity according to the purpose of the sources. For calibration sources with an activity greater than 1 MBq, which are exemplarily mentioned in ISO 2919, temperature, pressure, impact and penetration tests according to Class 2 as defined in the standard are required; a vibration test is not required. For the produced rod sources, which typically contain an activity significantly smaller than 1 MBq, these requirements were adopted because specific requirements for sources with activities of less than 1 MBq are not included in ISO 2919. The tests were performed on rod sources with ^88^Y because, due to the short half-life of this radionuclide, any possible release of activity from the rod sources in the course of the tests would not have had any long-term consequences. The activity of the rod sources was about 1000 Bq each. After each test, wet wipe tests with ethanol were conducted on the rod sources and the wipe-test swabs were measured in a specialized wipe-test counter for 1 min. All tests were performed with the same three sources in the order described below.

For the impact test, each rod source was positioned on a steel anvil. A cylindrical steel weight of 57 g with flat bottom was dropped on the rod source from a height of 1 m. For the puncture test, the same steel weight, but with a pointed bottom (a steel punch with 3 mm diameter in the bottom of the weight), was dropped also from a height of 1 m. The stipulated minimal masses are 50 g and 1 g, respectively. The falling motion of the weight was guided by three vertical metal bars in the layout of an equilateral triangle. In several subsequent tests, the middle of the side of the rod source as well as the faces with the plugs were exposed to the test.

For the temperature test, the rod sources were cooled using 8 kg of dry ice and kept at that temperature for 20 min. Additionally, they were heated to a temperature of 80 °C (the stipulated temperature) in a drying oven and kept there for 1 h. Both temperatures were checked with a Pt-100 resistance thermometer. The temperature in dry ice was measured as − 66 °C, which is well below the required temperature of − 40 °C.

For the external pressure test, the rod sources were put into a desiccator, which was evacuated to a pressure of 14 kPa using a rotary-vane pump. This pressure is below the stipulated pressure of 25 kPa. The rod sources were kept there for 5 min twice with a return to normal pressure in between. The pressure was checked with a pressure gauge that is part of the pump.

**Fig. 1:**
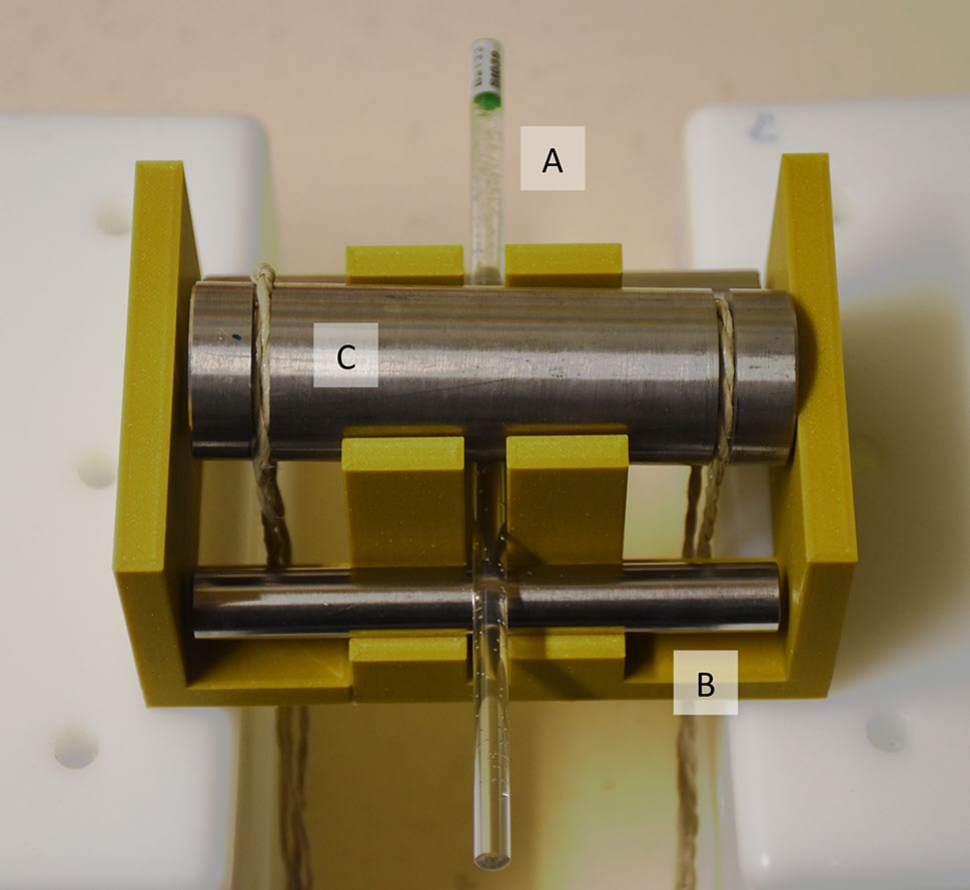
Setup for the bending test. The rod source (**A**) rests on two steel bars (steel bar in the front: **B**) and is bent by a third steel bar (**C**), which is pulled down using a specified mass

For the bending test, each rod source was positioned resting on two steel bars. A third bar acted a force onto the middle of the rod source so that the source was bent (Fig. 1). The force was controlled by hanging a mass from the third bar. The mass was gradually increased to check at which mass the rod sources were broken. The stipulated mass is 10.2 kg. This test is the type of bending test that is relevant for rigid sources with a length of more than 15 times their diameter according to Clause 7.7.1 of ISO 2919.

Additional to the tests according to ISO 2919, a rod source was sawn perpendicular to its long axis, the sawdust was removed from the source and the newly formed faces of the unsealed radioactive filling were wipe-tested.

## Results and discussions

### Overview

**Fig. 2:**
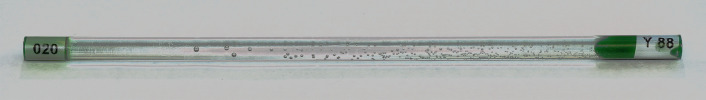
Produced rod source with a usual amount of bubbles

By now, about 900 rod sources with the following radionuclides were produced (Fig. 2):

- for the calibration of a whole-body counter: ^68^Ge, ^88^Y, ^133^Ba, ^241^Am, mixture of ^57^Co, ^60^Co, ^85^Sr, ^88^Y, ^109^Cd, ^137^Cs, ^139^Ce, ^203^Hg and ^241^Am;
- for research projects: ^90^Sr (for the measurement of secondary bremsstrahlung), ^252^Cf (for the measurement of secondary gamma radiation produced by neutron capture of hydrogen; Meisenberg et al. 2020);
- for the European Intercomparison of In-vivo Counters EIVIC 2020–2022: ^134^Cs and ^137^Cs, ^68^Ge and ^88^Y, ^133^Ba and ^152^Eu.

All radionuclides were dissolved in the acids HCl or HNO_3_, which are suitable solvents for most chemical elements. By now, no rod sources were produced with radionuclides dissolved in alkaline NaOH or in organic solvents or with isotopes of volatile chemical elements. Activities ranged from about 60 Bq to about 1,800 Bq (of ^88^Y) per rod source.

The density of the produced rod sources was 1.13 g/cm^3^ (5.3 g per source), compared to 0.86 g/cm^3^ (4.0 g) of the rod sources provided by the manufacturer of the phantom. Assuming that a brick is equipped with four rod sources, this alters the overall density of a brick only from 0.896 g/cm^3^ with the rod sources from the manufacturer to 0.901 g/cm^3^ with the produced rod sources (relative increase by 0.56%).

The material costs were about 1 € per rod source plus the costs for the radionuclide solution. On average, ten rod sources could be produced by one person per day.

Problems that occurred during the production of the rod sources caused a discard of about 5% of the sources. These problems comprised the following aspects:

- Accidental external contamination of the tubes with epoxy resin: even small contaminations were distinctly visible so that it was possible to discard the affected rod source immediately.
- Drift of the reading of the analytical balance: to determine the amount of radionuclide solution in the rod source, masses in the order of magnitude of 10–100 mg must be determined accurately. This required the usual precautions when using analytical balances, regular quality assurance of the balance, constant ambient conditions, and lack of air draught at the site of the balance. In particular, the mixing vessel needed to be earthed before each weighing to reduce any electrostatic charge.
- Failure of the mixture to harden: too much of the acid radionuclide solution caused the mixture of epoxy resin and hardener to fail to harden. This occurred to some extent at a radionuclide solution of 300 µl and entirely at 1000 µl.
- Drift of the activity of the rod sources over the course of production of a set: over the course of production of 160 rod sources, which typically took two months (for ^133^Ba and ^152^Eu sources), the rod activities tended to increase on average by 2.2%. This could have been caused by evaporation of the radionuclide solution. This problem can be avoided by an expeditious production of the rod sources and regular checks of the mass of the radionuclide solution.

Since the start of the production about 3 years ago, none of the produced rod sources showed any noticeable deterioration in quality. Only in rod sources containing ^252^Cf, a nuclide that features spontaneous fission, the number of bubbles in the filling increased, possibly caused by the accumulation of gaseous fission products. It is emphasised, however, that this did not compromise the quality of the sources.

### Quality-assurance measurements

**Fig. 3:**
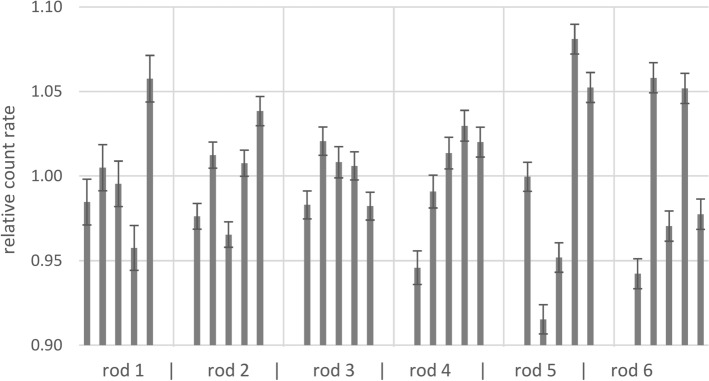
Count rates measured for each of five segments of six rod sources, relative to the arithmetic mean of the count rates of the segments of the respective source. Rod numbers are not the same as in Figs. 4, 5. Error bars represent two-sigma uncertainties of the counting statistics

Measurements of the spatial distribution of the activity along the rod sources were performed with six rod sources that were arbitrarily taken from the set of sources (Fig. 3). The relative double standard deviation of the five segments along each rod was 7.1% on average and 12% at most (rod 5 in Fig. 3). The maximal deviations of single segments from the average activity were − 8.5% (segment 2 of rod 5) and + 8.1% (segment 4 of rod 5). These deviations are sufficiently small for the application of the rod sources in phantoms, and did not significantly affect the quality-assurance measurements of the activities of the entire rod sources. So far it was not possible to identify the reasons why some segments showed particularly low or high activities.

**Fig. 4:**
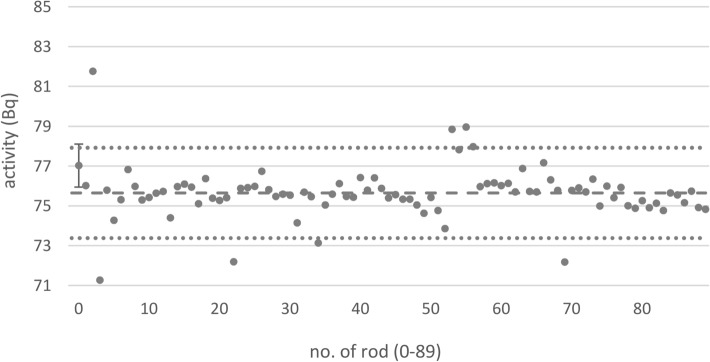
Activities of 90 single rod sources containing ^137^Cs calculated from the mass of the radionuclide sources. Dashed and dotted lines: arithmetic mean of the activities of those rod sources whose activities do not deviate by more than ± 3% of this mean, and boundaries of the ± 3% region, respectively. The uncertainty bar shown for one rod source is representative for all sources, and represents two sigma of the activity

The activities of 90 rod sources containing ^137^Cs were calculated from the mass of the radionuclide solution (Fig. 4). The activities ranged from 71.3 to 81.8 Bq with an arithmetic mean of 75.6 Bq and a relative double standard deviation of 3.4%. To reduce the deviation of the activities, sources were selected such that all of their activities ranged between − 3 and + 3% around their arithmetic mean. This resulted in a discard of eight sources (8.9%). The resulting arithmetic mean did not change (75.6 Bq) but the relative double standard deviation decreased to 1.8%.

**Fig. 5:**
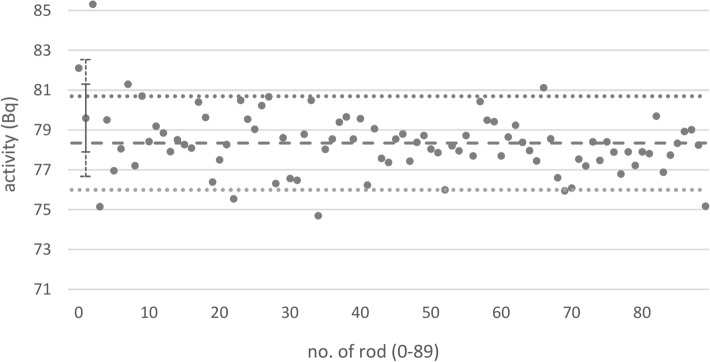
Same as Fig. 4, but with the ^137^Cs activities calculated from quality-assurance measurements using a gamma-spectroscopy detector. The uncertainty bars shown for one rod source are representative for all rod sources. Solid uncertainty bar: uncertainty due to the counting statistics of the measurement (uncorrelated between the single rod sources and thus indicating the variation of the results), dashed uncertainty bar: additionally taking into account the uncertainty of the calibration, which is a systematic contribution to the uncertainty and relevant for the comparison with the results from Fig. 4 but not for the comparison of the sources with each other)

The ^137^Cs activities were also calculated from gamma-spectroscopy measurements (Fig. 5). The activities ranged from 74.7 to 85.3 Bq with an arithmetic mean of 78.3 Bq (3.6% greater than the arithmetic mean of 75.6 Bq calculated from the mass of the radionuclide solution; see above). The relative double standard deviation of the values was 4.0%. After ten sources (11%) with activities outside a range of ± 3% around the arithmetic mean had been discarded, the arithmetic mean of the remaining sources did not change (78.3 Bq) but the relative double standard deviation decreased to 2.8%.

The uncertainties of the arithmetic means were 2.4 Bq (3.0%) for the gamma-spectroscopy measurements (based on the uncertainty of the calibration) and 0.8 Bq (1.0%) for the masses of the radionuclide solution. The confidence intervals of the arithmetic means of both methods overlap (upper limit of the mean activity based on the masses of the solutions: 76.4 Bq; lower limit of the mean activity based on the gamma-spectroscopic measurements: 76.0 Bq), showing that the results of both measurement methods are consistent with each other. The relative combined double uncertainty of the gamma-spectroscopy measurements of 3.7% (calculated from 3.0% uncertainty of the calibration and 2.1% of the uncertainty due to the counting statistics) can be taken as the uncertainty of the activity of these sources.

**Fig. 6:**
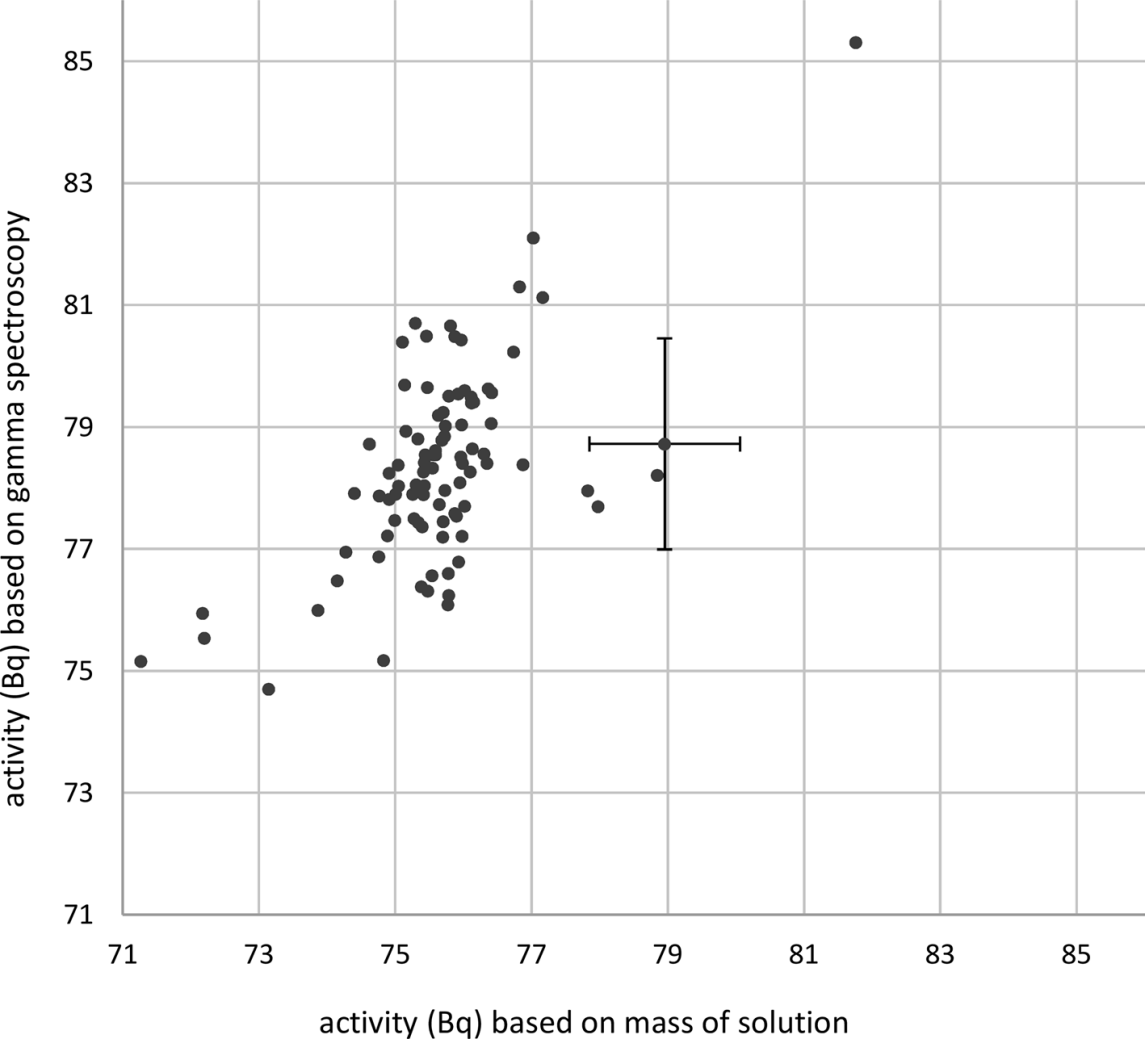
Comparison of the ^137^Cs activities of each rod source calculated from the masses of the radionuclide solution and those calculated from quality-assurance measurements. The uncertainty bars plotted for one rod source are representative for all rod sources

The activities based on the gamma-spectroscopy measurements show some correlation with those based on the masses of the radionuclide solutions (*R*^2^ = 0.35, Fig. 6).

### Classification as sealed radioactive sources

The impact test was passed without leakage and any visible damage to the rod sources; presumably, the rod sources would also have passed this test at the next level of severity (Class 3: 200 g from 1 m height) but this was not tested. The puncture test was passed without leakage but with superficial scratches in the PVC tubes. The temperature test was passed without leakage, any visible damage, and without softening of the tubes when they were warm; however, at slightly higher temperatures than 80 °C the PVC tubes will probably be adversely affected as PVC is expected to soften above about 80 °C. The external pressure test was passed without leakage and any visible damage. During the bending test the sources were broken at masses between 12 and 14 kg, thus they passed this test by only a narrow margin. In the additional wipe test of the newly formed faces of the unsealed filling no activity was detected on the swabs. This demonstrates that even after damage to the tube a release of radioactivity from the filling is unlikely. It was also found that the filling was firmly attached to the tube. The results mean that the sources can be certified as sealed radioactive sources according to ISO 2919, Class 2.

## Conclusion

The presented method is feasible to produce radioactive rod sources for the Saint-Petersburg brick phantom with only little specific requirements for the producing laboratory. This allows production of such sources for a variety of radionuclides required for the calibration of whole-body counters, which in turn is important for scientific research and interlaboratory comparisons. Because production of such sources turned out to be relatively easy, it can be done by users of whole-body counters themselves. This allows production of rod sources with short half-lives. Because the produced rod sources allow certification as sealed radioactive sources according to ISO 2919, they can even be used in radiation areas where only sealed sources are allowed. This facilitates provisions of operational radiation protection at whole-body counting laboratories. The robustness of the rod sources also improves their applicability for the calibration of mobile whole-body counters and for the shipment of the rod sources when they are exchanged between laboratories or used in interlaboratory comparisons. It is concluded that the method described here for the production of radioactive rod sources enhances and facilitates the use of the Saint-Petersburg brick phantom for the calibration of whole-body counters used for dose assessment after incorporation of radioactive substances.
